# Behavioral and neurophysiological taste responses to sweet and salt are diminished in a model of subclinical intestinal inflammation

**DOI:** 10.1038/s41598-020-74632-6

**Published:** 2020-10-19

**Authors:** David W. Pittman, Guangkuo Dong, Alexandra M. Brantly, Lianying He, Tyler S. Nelson, Schuyler Kogan, Julia Powell, Lynnette Phillips McCluskey

**Affiliations:** 1 Department of Psychology, Wofford College, Spartanburg, SC USA; 2 Department of Neuroscience and Regenerative Medicine, Medical College of Georgia at Augusta University, 1120 15th Street/CA-3016, Augusta, GA 30912 USA; 3 Department of Pathology and Laboratory Medicine, Medical University of South Carolina, Charleston, SC USA

**Keywords:** Taste receptors, Neuroimmunology

## Abstract

There is strong evidence for gut-taste bud interactions that influence taste function, behavior and feeding. However, the effect of gut inflammation on this axis is unknown despite reports of taste changes in gastrointestinal (GI) inflammatory conditions. Lipopolysaccharide (LPS), an inflammatory stimulus derived from gram-negative bacteria, is present in the normal GI tract and levels increase during high-fat feeding and gut infection and inflammation. Recordings from the chorda tympani nerve (CT), which transmits taste information from taste buds on the anterior tongue to the brain, previously revealed a transient decrease in sucrose responses in mice that ingest LPS during a single overnight period. Here we test the effect of acute or chronic, weekly LPS gavage on licking behavior and CT responses. Using brief-access testing, rats treated with acute LPS and mice receiving acute or chronic LPS decreased licking responses to sucrose and saccharin and to NaCl in mice. In long-term (23 h) tests chronic LPS also reduced licking responses to saccharin, sucrose, and NaCl in mice. Neurophysiological recordings from the CT supported behavioral changes, demonstrating reduced responses to sucrose, saccharin, acesulfame potassium, glucose and NaCl in acute and chronic LPS groups compared to controls. Chronic LPS significantly elevated neutrophils in the small intestine and colon, but LPS was not detected in serum and mice did not display sickness behavior or lose weight. These results indicate that sweet and salt taste sensitivity could be reduced even in asymptomatic or mild localized gut inflammatory conditions such as inflammatory bowel disease.

## Introduction

The gut environment modulates the function of distant neural systems^1–3^ including the gustatory system. Oral taste buds^4–6^ and central taste neurons^7^ respond to gut peptides that regulate feeding and satiety, but whether GI inflammation alters taste function is not known. Limited evidence suggests that gut inflammatory conditions such as inflammatory bowel disease are associated with changes in food intake and taste preference^8–12^ which can independently affect nutrition and health^13^. Lipopolysaccharide (LPS), an inflammatory component in the outer membrane of gram-negative bacteria^14^, is normally present in the GI tract but levels increase in GI infection^15–18^, gut inflammation (e.g. inflammatory bowel disease) in susceptible patients or animal models^19–21^, and a high-fat diet^22–26^. We previously demonstrated reduced neurophysiological sweet responses in mice that ingested LPS^27^. Specifically, sucrose responses recorded from the chorda tympani nerve (CT), which transmits peripheral signals from taste buds on the anterior tongue to the brain, transiently decreased seven days after a single overnight period of LPS ingestion. The reduction in sweet taste responses was dependent on the LPS receptor, toll-like receptor (Tlr)-4, and likely initiated in the gut rather than in taste buds although both sites express Tlr-4^28,29^. Taste bud expression of the sweet taste receptor subunits, T1r2 and T1r3, was transiently reduced by LPS ingestion in parallel with dampened sucrose responsivity at day 7 post-ingestion though restored to control levels at day 14^27^. Since a single exposure to LPS altered sweet taste function, in the current study we tested whether behavioral taste responses are also affected. We also asked whether changes in taste function and behavior are sustained when the gut environment is persistently altered with repeated LPS exposure as in chronic GI inflammatory and infectious conditions.

Here we report the effects of acute and weekly, chronic oral LPS gavage on behavioral taste responses using brief-access (15 s) and long-term (23 h) tests measuring licking responses to taste stimuli in mice and rats. Acute LPS treatment decreased licking to sucrose and saccharin in both mice and rats while chronic LPS diminished sweet and salt licking in mice. CT nerve responses to sweet and salt tastants also decreased in mice after acute and chronic LPS gavage. Though chronic LPS treatment elevated gut neutrophils, LPS did not leak into the circulation indicating that subclinical GI inflammation impacts taste function and behavior by activating a gut-taste axis.

## Results

We first tested whether a single, acute exposure to enteral LPS reduces behavioral taste responses in parallel with neurophysiological responses to sucrose^27^. In this study we administered LPS by gavage for more precise dosing, to bypass taste buds, and to avoid the ingestion of a palatable vehicle^27^. We also determined the effects of weekly, chronic enteral LPS on behavioral and neural taste responses. Immune responses to repeated LPS exposure become tolerized^30^, so it is possible that taste changes also weaken over time.

### Behavioral responses to sweet stimuli are reduced by enteral LPS gavage

**Figure 1:**
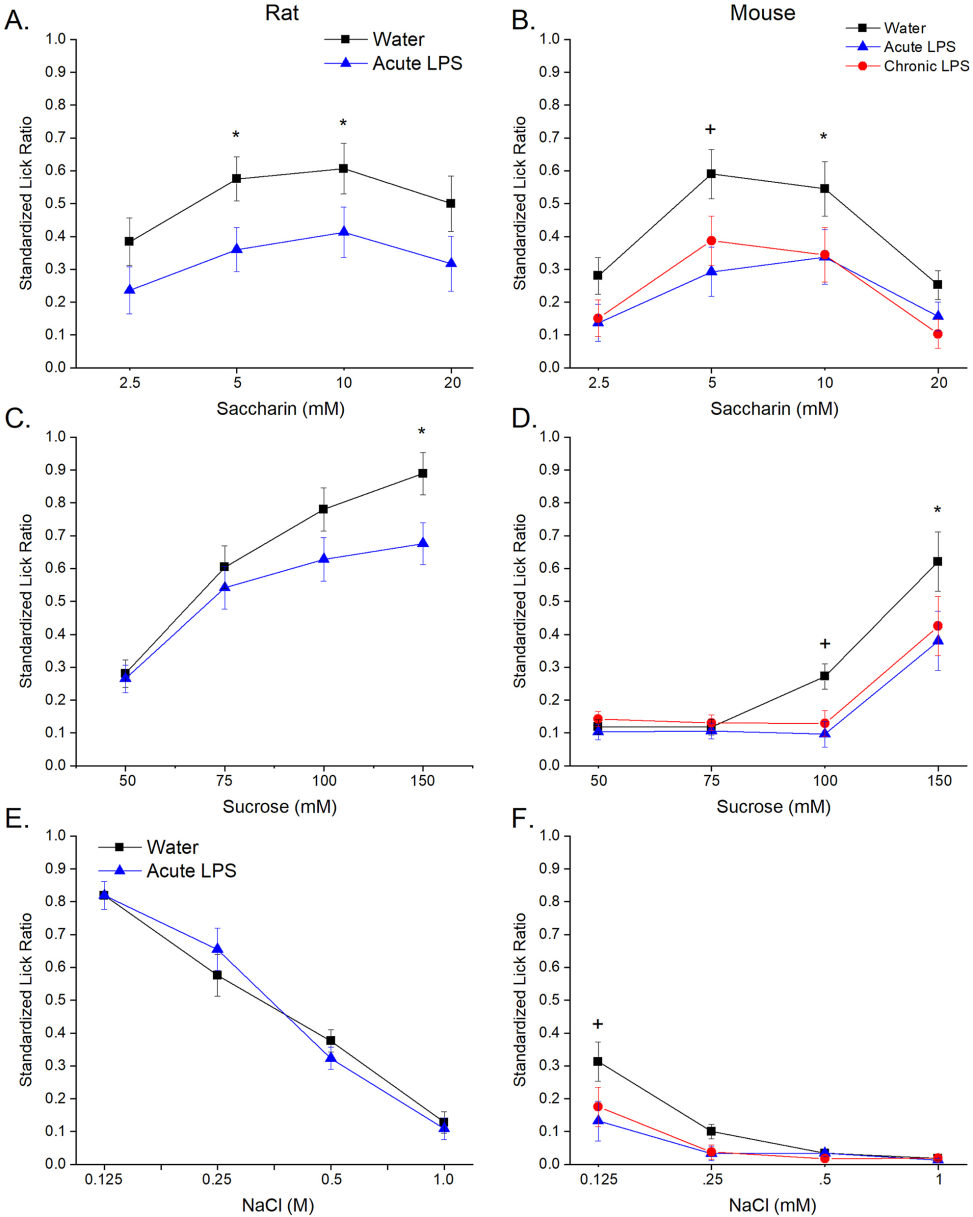
Average standardized lick ratios (± S.E.M.) during brief-access (30 s) trials for saccharin (**a** rats n = 13 / group; **b** mice n = 8 / group), sucrose (**c** rats; **d** mice) and NaCl (**e** mice). A star indicates a significant (*p* < .01) difference between control and LPS treatment(s); a plus indicates a significant (*p* < .05) difference between control and LPS treatment(s) for a specific concentration.

Licking to taste stimuli during brief-access trials (15 s duration) was measured following acute exposure to LPS in rats and both acute and chronic exposure to LPS in mice. As expected there were significant main effects for concentration across all taste stimuli for the brief-access testing in rats. Acute treatment with LPS reduced licking to both sweet tastants (Fig. 1a,c) with no significant effects on any other taste stimuli (NaCl, MSG, Quinine) (Fig. 1e, S1A, S1C). For licking responses to saccharin there was a significant main effect of experimental group [*F*(1,22) = 8.28, *p* = 0.009]; however, LPS treatment did not significantly decrease licking for all saccharin concentrations as post-hoc pairwise comparisons showed a reduction in licking for the LPS group at 5 mM (*p* = 0.004), and 10 mM (*p* = 0.001) concentrations. Similarly, licking to sucrose showed a significant main effect of experimental group [*F*(1,22) = 10.11, *p* = 0.004] with post-hoc pairwise comparisons identifying a reduction in licking for the LPS group at the highest concentration of 150 mM (*p* = 0.005).

There were significant main effects for concentration across all taste stimuli in brief-access testing in mice. There was a significant main effect of experimental group [*F*(2,21) = 4.99, *p* = 0.017] for licking to NaCl (Fig. 1f). Unlike the results for acute LPS treatment in rats, in the mice both acute and chronic LPS treatments reduced licking to the lowest tested concentration of 0.125 M NaCl (control vs. acute *p* = 0.041 and control vs. chronic *p* = 0.014). There was a significant main effect of experimental group [*F*(2,21) = 16.68, *p* < 0.001] for saccharin with post-hoc pairwise comparisons showing a reduction in licking for the LPS group at 5 mM (control vs. acute *p* = 0.013 and control vs. chronic *p* = 0.041), and 10 mM (control vs. acute *p* = 0.002 and control vs. chronic *p* = 0.004) concentrations (Fig. 1b). There was also a significant main effect of experimental group [*F*(2,21) = 7.47, *p* = 0.004] for sucrose with post-hoc pairwise comparisons showing a reduction in licking for the LPS group at 100 mM (control vs. acute *p* = 0.025 and control vs. chronic *p* = 0.038), and 150 mM (control vs. acute *p* = 0.001 and control vs. chronic *p* = 0.003) concentrations (Fig. 1d). Licking responses to MSG and QHCl were unchanged by acute or chronic LPS treatment (Fig. S1B,D).

**Figure 2:**
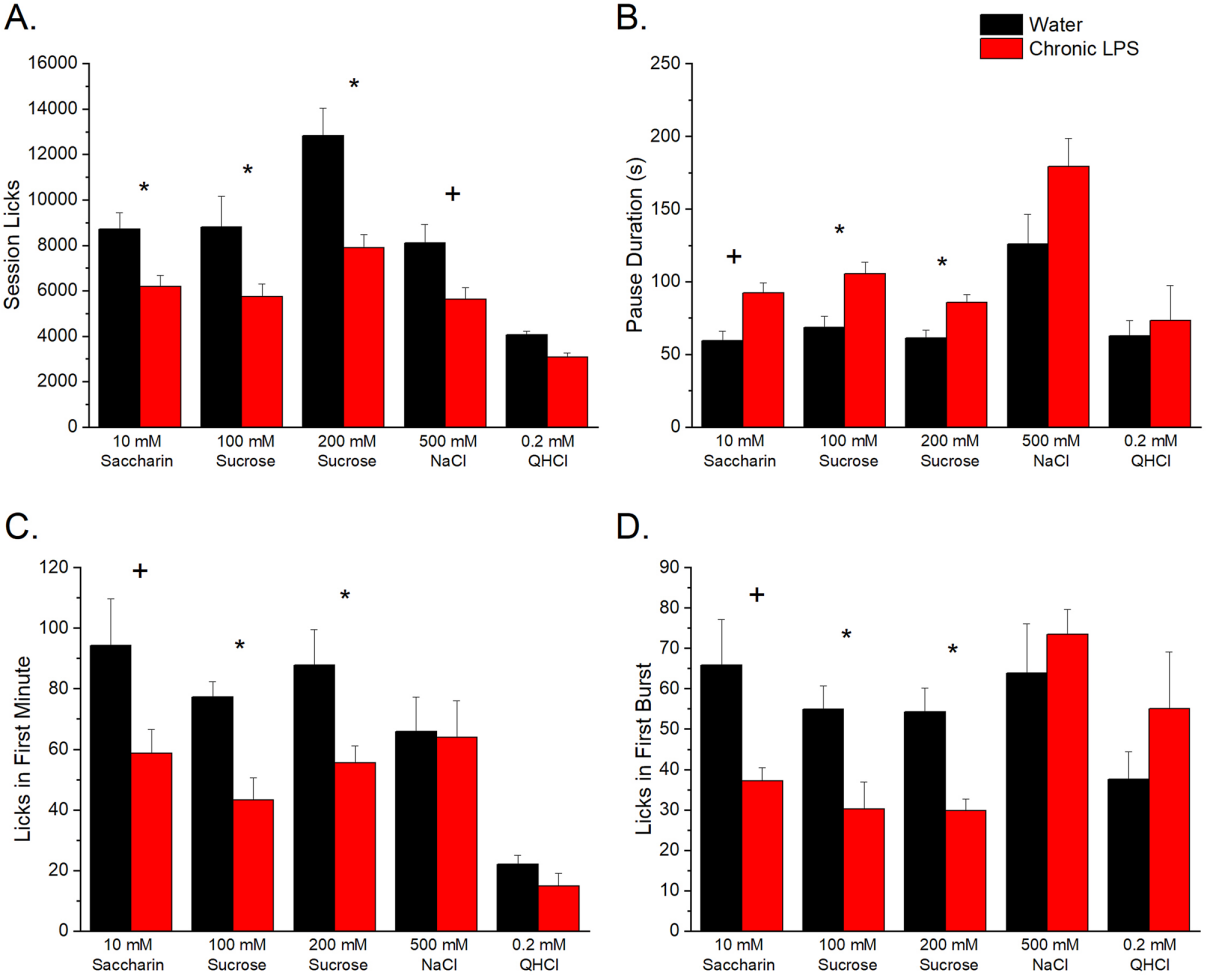
Average (± S.E.M.) session licks (**a**), pause durations (**b**), and licks in the first minute of testing (**c**), and licks in the first burst (**d**) during long-term (23 h) single bottle tests for saccharin, sucrose, NaCl, and quinine in mice. A star indicates a significant (*p* < .01) difference between control and LPS treatment; a plus indicates a significant (*p* < .05) difference between control (n = 8) and LPS (n = 8) treatment.

Patterns of licking across 23-h periods were measured following chronic exposure to LPS in mice. Long-term behavioral testing in mice revealed a significant effect of chronic LPS treatment to reduce the number of session licks for 10 mM saccharin [*t*(7) = 3.84, *p* = 0.006] and 100 and 200 mM sucrose [*F*(1,14) = 20.31, *p* < 0.001] along with a significant effect of concentration [*F*(1,14) = 7.99, *p* = 0.013] with 200 mM sucrose having more session licks (10,363 ± 674 S.E.M.) than 100 mM sucrose (7274 ± 731 S.E.M.)(Fig. 2a). The decrease in session licks for the sweet stimuli was accompanied by significant increases in the time spend paused from licking for 10 mM saccharin [*t*(7) = 3.12, *p* = 0.017] and both sucrose concentrations [*F*(1,14) = 17.76, *p* = 0.001] (Fig. 2b). The licks in the first minute of the session and the number of licks in the first burst are considered measures of the palatability of the solutions which are likely influenced by orosensory sensations such as taste rather than post-ingestive cues^31–34^ (Fig. 2c,d). Chronic treatment with LPS resulted in few licks in the first minute for 10 mM saccharin [*t*(7) = 3.53, *p* = 0.010] and both sucrose concentrations [*F*(1,14) = 14.27, *p* = 0.002]. Likewise, the number of licks in the first burst were reduced by chronic LPS treatment for 10 mM saccharin [*t*(7) = 3.11, *p* = 0.017] and both sucrose concentrations [*F*(1,14) = 13.94, *p* = 0.002].

As shown in Table 1, chronic LPS treatment affected several variables associated with post-ingestive cues but only for 200 mM sucrose. There were increases in the meal duration, number of bursts, and the average duration of the bursts for the LPS treatment group compared to the control group.

**Table 1:** Significant microstructural variables associated with post-ingestive cues for 200 mM sucrose.

Variable	Control Mean ± S.E.M	LPS Mean ± S.E.M	t-value	p-value
Meal Duration (s)	2600 ± 299	4728 ± 687	2.910	.023
Number of Bursts	44.9 ± 5.2	71.2 ± 8.1	2.582	.036
Burst Duration (s)	4.39 ± 0.49	2.98 ± 0.31	2.450	.044

Chronic LPS treatment also significantly reduced session licks for 0.5 M NaCl [*t*(7) = 2.48, *p* = 0.043] and decreased the number of meals [*t*(7) = 2.55, *p* = 0.038; control 10.8 ± 1.6 S.E.M., LPS 5.8 ± 0.9 S.E.M.] without significant effects on any of the other microstructural analysis variables (Fig. 2a–d). There was no effect on any of the microstructural analyses for 0.2 mM quinine.

### Neural taste responses to sweet and salt are reduced by acute and chronic enteral LPS

**Figure 3:**
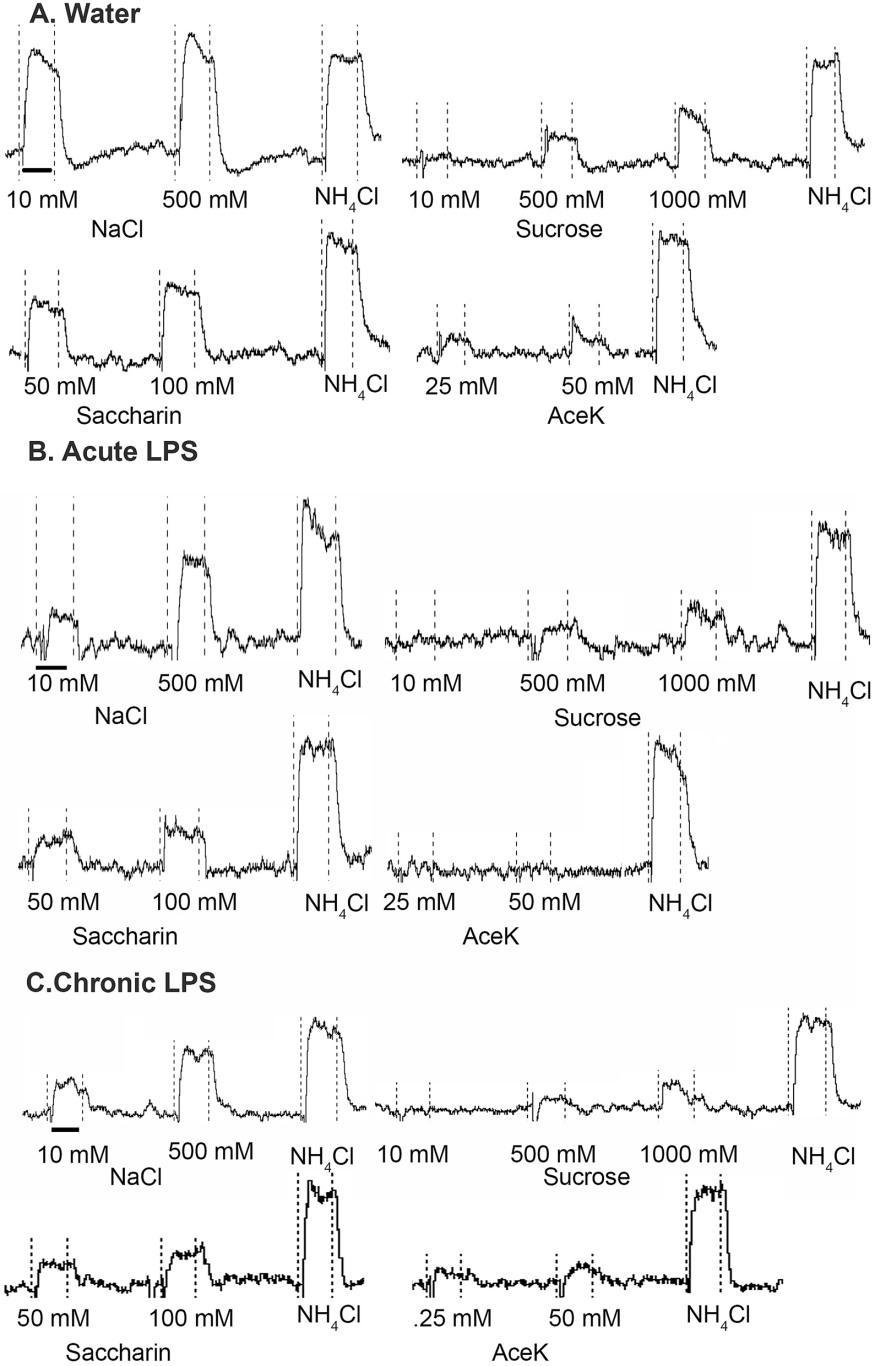
Representative integrated CT nerve response traces recorded at day 7 (water and acute LPS) or day 35 (chronic LPS) from mice gavaged with (**a**) water, (**b**) acute LPS, or (**c**) chronic LPS. Responses to sweet and salt tastants appeared smaller in LPS-treated mice compared to water-gavaged control mice. Responses to additional bitter, acid, and umami taste stimuli are not shown. Stimulus series are bracketed by NH_4_Cl responses (one shown per series). Dotted vertical lines indicate stimulus application and rinse. The scale bar under the first response for each mouse represents 20 s.

**Figure 4:**
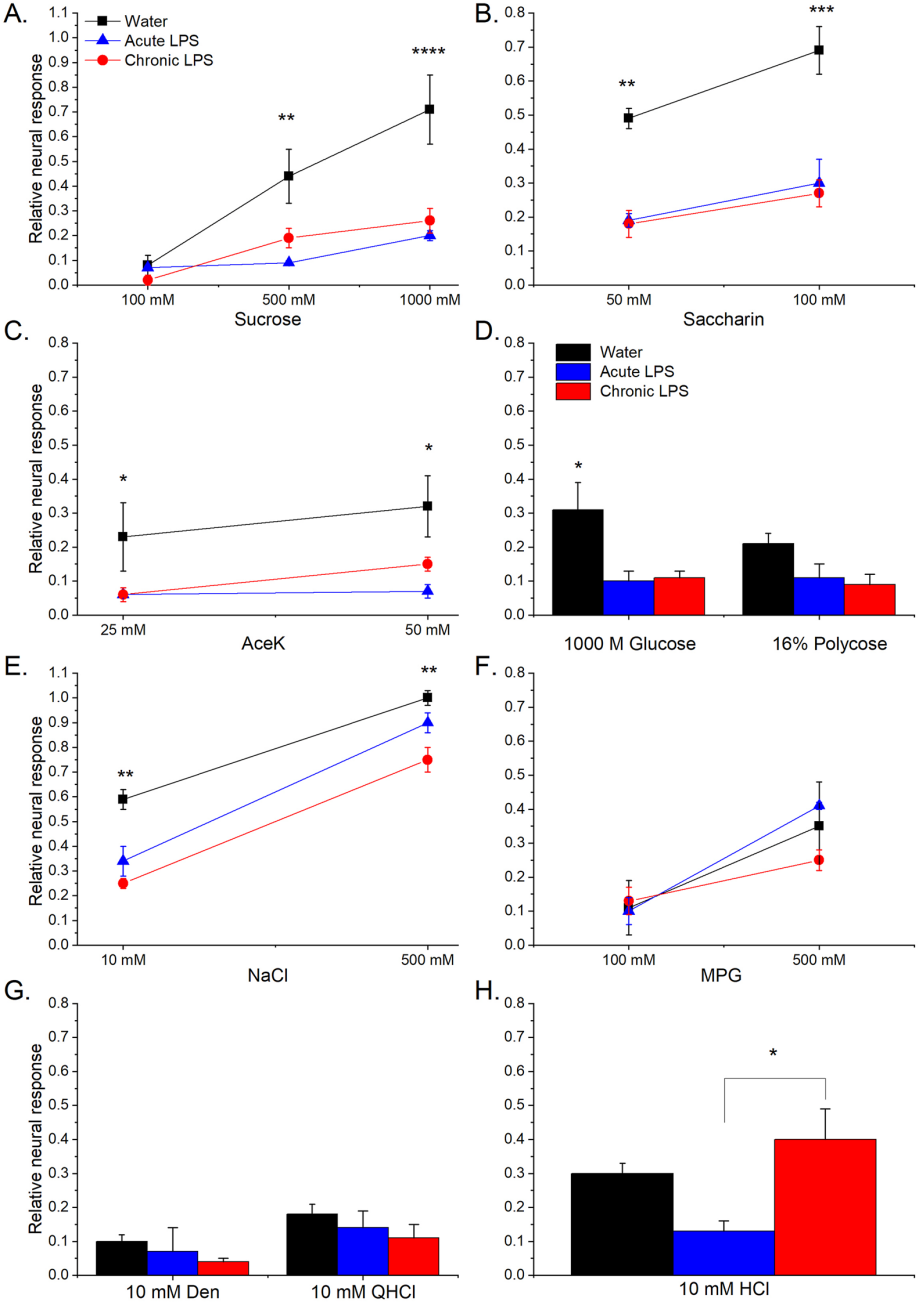
Mean (± S.E.M.) CT responses in mice gavaged with acute or chronic LPS vs. control mice. Mice were gavaged with water (n = 3), acute LPS (n = 5) or chronic LPS (n = 5). Both acute and chronic LPS significantly reduced neural responses to (**a**) sweet/starch stimuli, including 500 and 1000 mM sucrose, both concentrations of saccharin (**b**), 25 mM aceK in the acute LPS group and 50 mM aceK (**c**), and glucose (**d**) but not to the starch, polycose (**d**). (**e**) Neural responses to 10 mM NaCl were reduced by both acute and chronic LPS, but only chronic LPS significantly decreased responses to 500 mM NaCl. Responses to the umami stimulus, MPG (**f**), and bitter stimuli, denatonium and QHCl (**g**), were not significantly altered by acute or chronic LPS. Acute enteral LPS lowered responses to the acid stimulus, HCl, compared to the chronic LPS but not water-treated group. *p < .05; **p < .001; ***p < .0001. Actual *p* values are provided in the Results.

Examples of CT response traces from mice gavaged with water, a single dose of LPS, or weekly LPS are shown in Fig. 3. There were significant main effects of LPS treatment [*F*(2,10) = 15.36; p < 0.0009], stimulus concentration [*F*(2,20) = 52.36; p < 0.0001] and a significant interaction effect [*F*(4,20) = 10.23; p = 0.0001] on neural responses to sucrose (Fig. 4a). Specifically, responses to 500 mM sucrose were lower following acute (*p* = 0.0001) and chronic (*p* = 0.005) LPS groups compared to controls. Responses to 1000 mM sucrose were also lower in both acute and chronic LPS groups (*p* < 0.0001). As shown in Fig. 4b, CT responses to saccharin were significantly affected by acute and chronic LPS treatment [*F*(2,10) = 17.50; *p* = 0.005] and stimulus concentration [*F*(1,10) = 22.82, *p* = 0.0007] with reduced CT responses to 50 mM (*p* = 0.002) and 100 mM saccharin (*p* < 0.001) compared to water-gavaged control mice. Acesulfame potassium (aceK) responses were similarly reduced by LPS treatment [*F*(2,11) = 6.39, *p* = 0.01] and concentration [*F*(1,9) = 11.54, *p* = 0.0008] (Fig. 4c). Acute LPS decreased neural responsivity to 25 mM aceK, (*p* = 0.047) while both acute (*p* = 0.003) and chronic (*p* = 0.032) treatment diminished responses to 50 mM aceK compared to control mice. There was a significant main effect of treatment on responses to 1 M glucose [*F*(2,10) = 7.46, *p* = 0.01] with decreases in the acute (*p* = 0.016) and chronic (*p* = 0.02) LPS groups vs. controls (Fig. 4d). While there was a significant main effect of treatment on polycose responses [*F*(2,10) = 4.23, *p* = 0.047], groups were not significantly different in post-tests.

In addition to the changes in sweet responses, there were significant effects of treatment [*F*(2,10) = 16.21; *p* = 0.0007] and stimulus concentration [*F*(1,10) = 190.3; *p* < 0.0001] on CT responses to NaCl (Fig. 4e). Specifically, neural responses to 10 mM NaCl were decreased by acute (*p* = 0.005) and chronic (*p* = 0.0002) LPS (Fig. 4b). However, CT responses to 500 mM NaCl were significantly reduced only by chronic LPS (*p* = 0.004). Neither acute nor chronic LPS significantly affected mean CT responses to the umami stimulus, MPG (Fig. 4f) or the bitter stimuli, denatonium and quinine (Fig. 4g). There was a significant effect of treatment on acid responses [*F*(2,10) = 4.73; *p* = 0.036] (Fig. 4h) but the difference was in the acute vs. chronic LPS groups (*p* = 0.037). In sum, neurophysiological taste responses to sweet and salt stimuli were inhibited by acute and chronic enteral LPS, though repeated treatment had broader effects on NaCl responses.

### Enteral LPS stimulates gut neutrophil responses but does not enter the circulation

**Figure 5:**
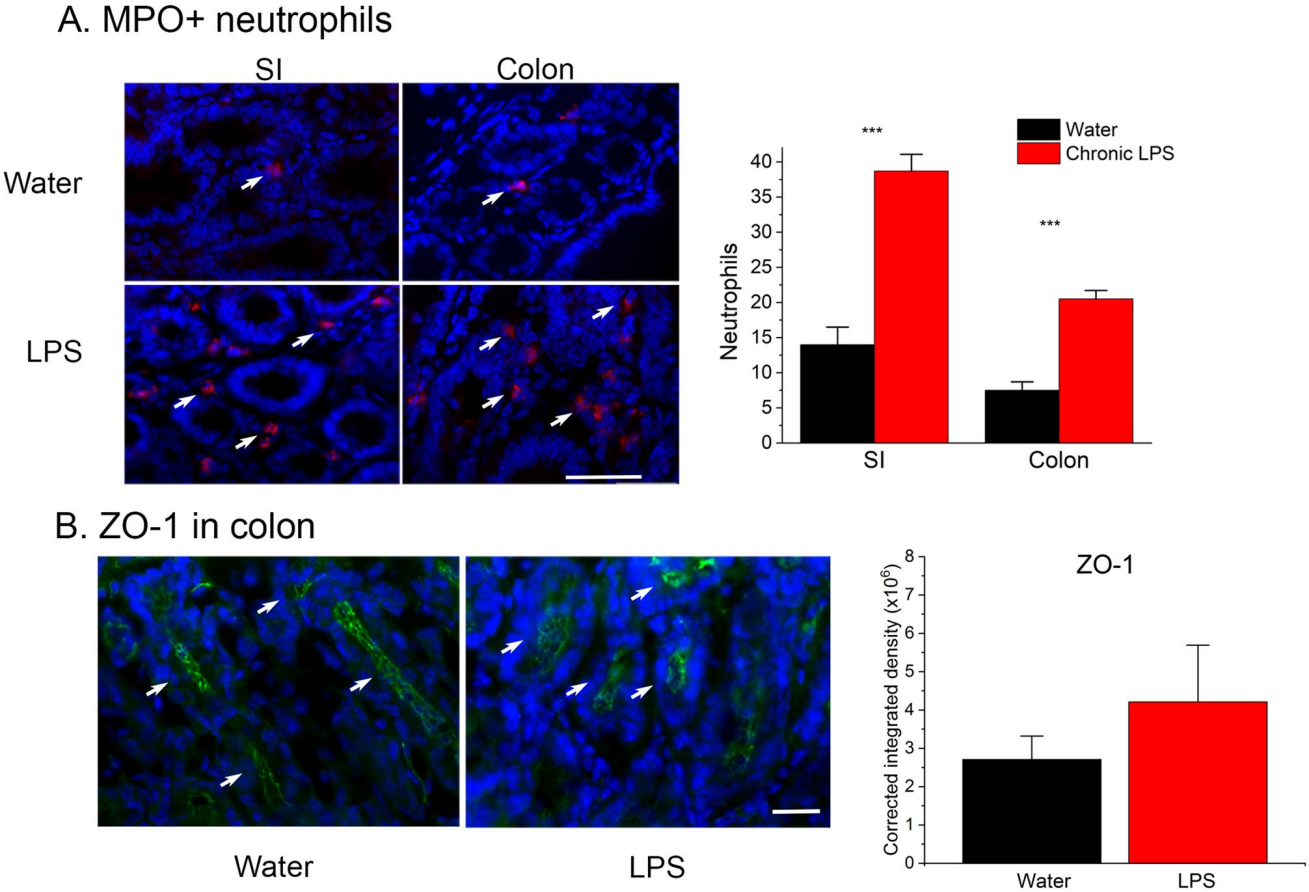
Chronic enteral LPS elevates gut neutrophils but does not alter ZO-1 expression. (**a**) MPO-positive neutrophils (red; arrows) were more numerous in the small intestine and colon of mice gavaged weekly for five weeks with chronic LPS (n = 4) compared to water (n = 4). Neutrophils / standard area (± S.E.M.) were significantly elevated in both gut regions in the chronic LPS group compared to the control group. (**b**) Colonic ZO-1 expression (green, arrows) appeared similar following chronic LPS (n = 4) or water gavage (n = 4). ZO-1 expression was not significantly different between groups. ***p < 0.0001. See text for actual *p* values. Scale bars = (**a**) 20 µm or (**b**) 35 µm.

We counted immunofluorescent, myeloperoxidase (MPO)-positive neutrophils in the small intestine and colon as a measure of GI inflammation. Significantly more neutrophils were present in the small intestine [*t*(7) = 6.373, *p* = 0.0004] and colon [*t*(6) = 7.66, *p* = 0.0003] of chronically treated LPS mice compared to water-gavaged controls (Fig. 5a). Since the gut becomes inflamed with this treatment and thus potentially leaky, we tested whether LPS enters the circulation which could elicit systemic inflammation. However, serum LPS was below the limit of detection in chronic LPS-treated [mean optical density (O.D.) 0.099 ± S.E.M. 0.004] and water-treated (mean O.D. 0.119 ± 0.01) mice compared with positive control serum (range over increasing LPS dilutions = 0.30, 0.57 and 0.77 O.D.). The absence of systemic LPS is consistent with our finding that colonic expression of the tight-junction protein, zona occludins (ZO)-1, as a measure of gut leakiness^35^ is not significantly altered by chronic LPS (Fig. 5b; *p* > 0.05).

### Expression of taste transduction genes is unchanged by chronic LPS

**Figure 6:**
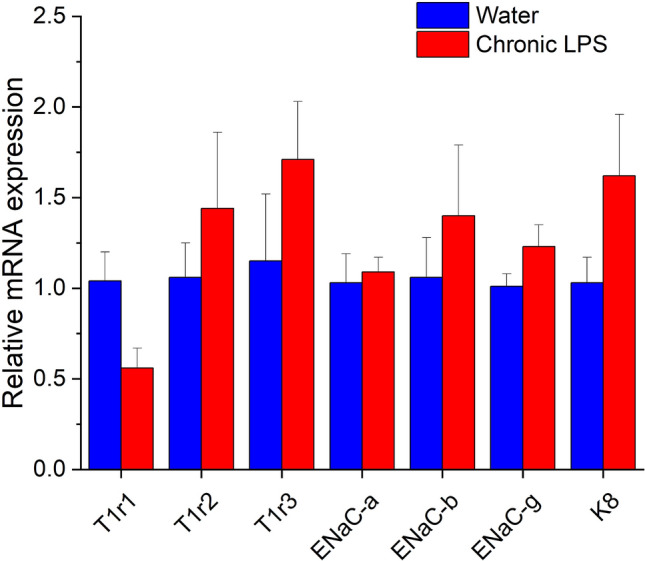
Chronic enteral LPS did not alter the expression of taste genes. Quantitative real-time RT-PCR was used to compare the expression of the umami and sweet taste receptor subunits, T1r1, T1r2, and T1r3; the ENaC (i.e. SCNN1)-a, -b, and -g subunits, and the taste receptor cell marker, K8, in anterior lingual epithelium. Expression levels were normalized to GAPDH. There were no significant differences in the chronic LPS group (n = 4) versus water-gavaged controls (n = 4).

Previously we reported that acute ingested LPS decreased T1r2 and T1r3 mRNA levels at day 7 though expression returned to control levels at day 14 after treatment. However, it is possible that T1r2/3 expression levels fell again after chronic enteral LPS. We also tested whether the expression of salt receptor subunits decreased in parallel with neural and behavioral responses. As shown in Fig. 6, however, umami subunit T1r1, sweet taste receptor subunits T1r2 and T1r3, and ENaC-alpha, beta, and gamma mRNA expression in the lingual epithelium was similar between groups (*p* > 0.05 for all genes). The stable expression of the taste receptor cell marker, keratin-8 (K8), in the control and experimental groups indicates that LPS does not cause taste dysfunction through taste receptor cell loss.

### Enteral LPS does not induce sickness behavior or alter food intake or body weight

Changes in neurophysiological and behavioral taste responsivity could be initiated by decreased food intake and body weight. However, weekly enteral LPS had no significant effect on body weight over 8 weeks though mice gained weight over time in both groups as expected (Fig. S2) [*F*(44,440) = 14.98; *p* < 0.0001]. Neither treatment nor time had any significant effect on food intake (Fig. S2 Furthermore, we did not observe sickness behavior in the control or chronic LPS groups at any time (not shown).

## Discussion

We report that acute and chronic enteral LPS altered behavioral and neurophysiological taste responses in generally similar ways in rodents. Acute LPS decreased licking responses to natural and artificial sweet stimuli in brief-access tests in both rats and mice. Chronic LPS, tested in mice, revealed similar decreases in both brief-access and long-term licking responses to sweet stimuli. Reduced CT responses to sweet stimuli supported these behavioral changes, though significant neurophysiological effects emerged at higher concentrations of sucrose and saccharin as expected given the higher sensitivity of behavioral tests. Importantly, behavioral and neurophysiological changes elicited by enteral LPS were maintained rather than weakening over time, or tolerizing, as observed in inflammatory responses to systemic LPS^30,36^. Sustained taste changes are consistent with our finding that chronic LPS treatment provokes local, gut inflammatory responses but does not enter the systemic circulation at this dose.

In addition to sweet-specific changes we demonstrate reduced neural and behavioral responses to salt in LPS-treated mice. Acute and chronic LPS reduced licking responses to NaCl in brief-access behavioral tests and neurophysiological CT recordings, though at different concentrations. The effect occurred at 125 mM in brief-access behavioral tests, at 10 mM and 500 mM NaCl in CT recordings, and at 500 mM NaCl in long-term behavioral tests. The divergence in responses to 500 mM NaCl is not surprising since higher concentrations of NaCl are aversive to mice^37,38^, likely overriding significant reductions in neural salt taste transmission to the CNS. However, session licks were decreased in long-term behavioral tests in mice, which only had access to 500 mM NaCl. Acute LPS effects also diverged across species since licking responses to 125 mM NaCl were significantly reduced in mice but not rats in brief-access experiments. However, rats may have demonstrated changes in salt tests in further long-term, neurophysiological, and chronic LPS studies. We note that the number of licks in brief-access tests was far greater in rats, indicating their superiority as a rodent model of taste behavior though less amenable to genetic manipulation.

Short-term, brief-access tests in mice reflect taste-guided behavior while long-term tests are also affected by post-ingestive cues^39^. In long-term tests, chronically LPS-treated mice consumed less 200 mM sucrose at a slower rate than control mice, with increased meal duration and shorter but more numerous bursts with longer pauses between each burst. This suggests that mice were less motivated by the decreased palatability and decreased post-ingestive feedback pathways that normally stimulate appetitive behaviors. It is unlikely that mice reduced licking because of malaise since sickness behaviors were absent and food intake was maintained in LPS-treated mice, consistent with the sequestration of LPS in the gut. Enteral and intracolonic^40^ LPS induce local intestinal inflammation in contrast to highly proinflammatory systemic LPS which is used to model sepsis^41^ and has distinct effects on taste buds^42^ and CT responses^43^.

Acute enteral LPS reduced CT responses to sucrose, saccharin, AceK and glucose in contrast to the limited effects of acute ingested LPS on sucrose in a previous study^27^. The dose of LPS used here was based on our previous work, though more precise dosing may explain the broader effects on sweet responses. Acute enteral LPS also reduced CT responses to 10 mM NaCl while both 10 mM and 500 mM were decreased by chronic LPS. However, chronic treatment did not alter T1r2, T1r3, or ENaC subunit mRNA expression in lingual epithelium. We previously reported a transient downregulation of T1r2 and T1r3 expression at d7 after acute, ingested LPS followed by restoration of control levels of transcripts at day 14^27^, indicating that distinct mechanisms underlie extended neurophysiological changes following chronic LPS. Sweet and salt transduction elements could be regulated post-transcriptionally, receptor function could be modulated independently of receptor expression, or alternative sweet and salt transduction pathways or downstream signaling genes could be targets of enteral LPS.

What lies upstream from changes in taste function and behavior? Since LPS did not exit the GI tract to enter the circulation, a direct effect of LPS on taste buds through Tlr4^28^ is unlikely. Instead, the gut-taste axis could be mediated by hormones altered during gut inflammation or a dysregulated GI environment. Many gut hormones also bind their receptors on taste buds to regulate taste function^4,5,44^. For example, LPS activation of gut toll-like receptor 4 (Tlr4) drives the release of glucagon-like peptide-1 (GLP-1), serotonin, and peptide YY (PYY) by enteroendocrine cells^45,46^. Each of these hormones affects taste^5^, though the specific effects of LPS on sweet and salt responses narrow potential signaling intermediates. Candidates include sweet modulators such as leptin, endocannabinoids, and GLP-1, and insulin and ghrelin which affect salt taste^4,5,7^. Parallel changes in neural and behavioral responses indicate that taste buds or CT fibers are the primary targets for LPS though we cannot rule out parallel gut-brain signaling.

Taste is critical for nutrition yet the effects of gut inflammation on this sensory system are poorly understood^47,48^. Reduced neural and behavioral responses to sweet and salt indicate that the taste system is highly sensitive to intestinal and colonic inflammation induced by a relatively low weekly dose of enteral LPS. Our results suggest that hypoguesia and decreased sweet and salt intake could influence nutrition even in patients with asymptomatic or mild Crohn’s disease, ulcerative colitis, and *Helicobacter pylori* infection^20,49,50^. More than 85% of patients with Crohn’s disease and ulcerative colitis, together known as inflammatory bowel disease (IBD), are malnourished due to changes in food intake and absorption^51–53^. IBD patients also demonstrate reduced taste sensitivity to multiple tastants^8,11,12,54^, and higher sweet consumption has been reported in subjects with Crohn’s disease^9,10^. The enteral LPS model described here will be useful to dissect genetic and cellular mechanisms responsible for taste changes in the waning phase of chronic gut inflammatory conditions.

## Materials and methods

### Animals and LPS treatments

Adult female C57BL/6 J mice (Jackson Laboratory) or Sprague–Dawley rats (Charles Rivers Laboratory) were 6–10 weeks old at the time of treatment. We used female mice consistent with our previous study on ingested LPS^27^. Mice were housed in a SPF barrier facility on a 12:12-h light: dark cycle with lights on at 6:00 am. Unless otherwise specified mice were provided free access to rodent chow (Purina) and tap water. LPS (*E. Coli* O26:B6; #L8274 Sigma) was freshly prepared at a dose of 2.8 mg/kg body weight in 100 µl sterile, endotoxin-free distilled water and administered by oral gavage into the stomach, bypassing the oral taste buds, with sterile, flexible needles (#9924B, Cadence Science) attached to a syringe. This dose was based on the amount of LPS voluntarily consumed in our previous study^27^, and is administered in a volume of approximately 25% of the murine stomach capacity. Control groups were gavaged with sterile, endotoxin-free, distilled water.

Animals receiving “acute LPS” were given a single dose at day 0 prior to initiating neurophysiological or behavioral experiments at day 7 as in previous work^27^. “Chronic LPS” groups were gavaged at day 0 and again weekly before beginning behavioral experiments at day 35 after the initial treatment. Pilot neurophysiological results demonstrated similar results at day 21 and 35 after weekly LPS gavage so the 3–4 week period was used for “chronic LPS” treatment for all additional experiments. Additional groups of control (n = 6) and chronically treated LPS mice (n = 6) were gavaged weekly with LPS or water for 7 weeks and body weight and chow consumption (corrected for spillage) measured daily for 8 weeks. Mice were gavaged and body weight and food intake measured between 8:00–11:00 am. We also observed mice daily for the following signs of sickness behavior: changes in body position (hunched back, prone, or lying on side), sustained eye closure, piloerection, dragging or elevated tail position, or failure to respond to stroking of the back^55^. Animal procedures followed National Institutes of Health guidelines and were approved by the Augusta University or Wofford College Institutional Animal Care and Use Committees.

### Brief-access behavioral testing

Licking responses to brief-access (15 s trials) taste stimuli were measured in rats (control n = 13, acute LPS n = 13) and mice following LPS treatment (control n = 8, acute LPS n = 8, chronic LPS n = 8). Three days prior to brief-access testing, all animals were placed and maintained on a 23 h water restriction schedule and trained to lick to water presentations in the MS-160 gustometer (DiLog Instruments). During testing of aversive stimuli (NaCl, quinine) rats were placed on 23 h water restriction prior to the testing day. During testing of appetitive stimuli [sucrose, saccharin, monosodium glutamate (MSG)] rats had ad lib food and water while mice needed food restriction with *ad lib* water 24 h prior to testing to motivate their licking behavior. All taste stimuli were mixed daily. Using a counterbalanced design, animals were tested for two consecutive days with have the animals tested with the aversive stimuli array and half receiving the appetitive stimuli array. During each daily test session, four concentrations of each tastant in the appetitive or aversive array along with water were presented in pseudorandom order once each in three blocks. Data for the three presentations of each stimulus were averaged for each test session. Each lick, average ILI, and the latency until the first lick were recorded. There were 10 s intervals between trials and per trial, animals had 30 s to initiate a lick followed by a 15 s trial duration after the first lick.

All brief-access licking data were converted to a standardized lick ratio^56,57^ which accounts for individual differences in baseline “local” (intraburst) lick rates independent of motivational state. The standardized lick ratio was calculated by dividing the average licks for each stimulus by each subject’s maximum potential lick rate. The maximum potential lick rate was determined by dividing the duration of the stimulus trial by the average interlick interval (> 50 ms and < 200 ms) recorded during water training tests, prior to the experiments. An analysis of interlick intervals did not reveal any significant group differences between control, acute LPS, and chronic LPS treatments (all *p* n.s.).

### Long-term behavioral testing

Licking behavior was measured over 5 consecutive 23-h periods during single bottle test sessions in the AC-108 contact lickometer (DiLog Instruments, Tallahassee, FL) following control (n = 8) or chronic LPS treatment (n = 8). Ad libitum food was available throughout the testing. Five tastants (10 mM saccharin, 100 mM sucrose, 200 mM sucrose, 500 mM NaCl, 0.2 mM quinine-HCl) were presented in a counterbalanced order during each 23-h test session. The contact lickometer recorded the time and duration of each lick during the 23-h test session at a resolution of 1 ms.

The session licks for each mouse during the 23-h test session were grouped into meals initiated by 5 licks within 1 s and terminated by a pause of 600 s^34^. The licks for each meal of the test session were subjected to a microstructure analysis in order to examine whole-meal measures (meal lick count and meal duration), intra-meal licking patterns (number of bursts, size of bursts, mean burst duration, mean pause duration, licks in the first minute, and licks in the first burst) to provide analysis of variables related to taste motivated licking and post-ingestive feedback motivated consumption. The average of each variable per meal were then averaged across all meals in the 23 h test session. Licking bursts were defined by a 1-s pause in licking resulting in a cumulative count of number of bursts per meal. Mean burst size is the total number of licks in the meal divided by the number of bursts in the meal. Mean burst duration is the average length of time for each licking burst within the meal. Mean pause duration is average length of time from the termination of a burst to the initiation of the next licking burst within a defined meal. Licks in the first minute of the meal along with licks in the first burst of a meal are considered consumption patterns motivated by afferent taste input as opposed to post-ingestive influences.

### Neurophysiology

CT nerve recordings were performed as previously described^27^ in mice gavaged with water (n = 3), acute LPS (n = 5), or chronic LPS (n = 5). Mice were anesthetized with ketamine (80 mg/kg) plus xylazine (10 mg/kg) i.p. and body temperature maintained at 36–39 °C. Supplemental anesthetic was injected as needed. Mice were tracheotomized and the hypoglossal nerves sectioned bilaterally to stop tongue movement. The right or left CT nerve was dissected using a lateral approach and placed on a platinum electrode with the indifferent electrode in nearby muscle. Neural activity was amplified (Grass Instruments), integrated with a time constant of 1.5 s, and the summated signal monitored with PowerLab hardware and software (AD Instruments).

We stimulated the anterior tongue with sucrose (100, 500 and 1000 mM), saccharin (50 and 100 mM), aceK (25 and 50 mM), 1.0 M glucose, 16% polycose, monopotassium glutamate (MPG; 100 and 300 mM), 10 mM QHCl, 10 mM denatonium, and 10 mM HCl mixed in distilled water. Approximately 1 ml of each stimulus was applied to the anterior tongue at room temperature using a syringe. After 25 s the tongue was rinsed with distilled water for ≥ 1 min. We calculated CT nerve response magnitudes by measuring the height of the summated, integrated traces at 20 s after stimulus application and subtracting the baseline. Test responses were expressed relative to mean responses to 500 mM NH_4_Cl applied at the beginning and end of the stimulus series. Only series bracketed by NH_4_Cl responses that deviated by ≤ 10% were included in analyses. At the end of the recording deeply anesthetized mice were euthanized by cervical dislocation and tissues collected for further analyses.

### Limulus amebocyte lysate (LAL) assays

Serum LPS was quantified in triplicate from mice chronically treated with water (n = 4) or LPS (n = 4) according to the manufacturer’s instructions (Chromogenic LAL Endotoxin Assay Kit, #L00350, Genscript) using an EL800 plate reader (Bio-Tek Instruments) with absorbance at 540 nm. Positive control wells with serum containing three increasing concentrations of LPS were also run in triplicate. The limit of detection for the kit is 0.01–1 EU/ml.

### Quantitative real-time PCR (qPCR) analysis

Mice were euthanized as above following chronic gavage with water (n = 4) or LPS (n = 4) and lingual epithelia harvested by collagenase treatment and manual dissection as previously reported^27^. Total RNA was extracted from the lingual epithelium anterior to the intermolar eminence using the Rneasy Fibrous Tissue Mini kit (Qiagen) and reverse transcribed using the QuantiTect Reverse Transcription kit (Qiagen)^27^. The cDNA was processed for real-time PCR using the QuantStudio 3 real-time PCR system (ThermoFisher). Primer pairs for taste genes are shown in Table 2. The 2-ΔΔCT method was used to express signal from the LPS group relative to the control group^58^.

**Table 2:** Sequences of primer pairs used for real-time RT-PCR.

Gene GenBack accession #		Sequence 5′-3′	Size (bp)
T1R1(NM_031867.2)			226
	Forward primer	TGGCAGCTATGGTGACTACG	
	Reverse primer	CAGCACCACAGACCTGAAGA	
T1R2(NM_031873.1)			212
	Forward primer	GCACCAAGCAAATCGTCTATCC	
	Reverse primer	ATTGCTAATGTAGGTCAGCCTCGTC	
T1R3(NM_031872.2)			100
	Forward primer	ACTACATACTGGGCGGGCTA	
	Reverse primer	GGTGAGAACCTGTTGCACGG	
ENaCa(NM_011324.2)			80
	Forward primer	CAGACTTGGACCTTTGACAAGGA	
	Reverse primer	ACTTCTCTGTGCCTTGTTTATATGTGTT	
ENaCb(NM_011325.2)			82
	Forward primer	GGCTAGAGTCCCTCTCCTGGTT	
	Reverse primer	GCCAACTAGGGCAAGGATTCT	
ENaCg(NM_011326.3)			166
	Forward primer	ACAAAAAGCTCAACAAGACTGACC	
	Reverse primer	TCGATGACACAGACGACCGA	
Krt8(NM_031170.2)			130
	Forward primer	TCTTCTGATGTCGTGTCCAAGTG	
	Reverse primer	GATCCTCGGACGGGTCTCTAG	
GAPDH(NM_001289726.1)			81
	Forward primer	GGAAGGGCTCATGACCACAG	
	Reverse primer	TCACGCCACAGCTTTCCAG	

### Immunofluorescence and image analyses

Colon and small intestine (SI; duodenum through ilium) from LPS (n = 4) or water treated (n = 4) mice were mounted in OCT (Fisher Scientific) as “Swiss rolls”^59^, cryosectioned at 8 µm, fixed with 0.4% paraformaldehyde and incubated in a rabbit anti-myeloperoxidase antibody (MPO; 1:100; ab9535, Abcam) or ZO-1 (1:100, #61-7300, Invitrogen) for 2 h at room temperature followed by goat anti-rabbit Alexa Fluor 594 secondary antibody (1:1000; Jackson ImmunoResearch Laboratories) for 1 h at room temperature before counterstaining with DAPI (Life Technologies). Quantification of MPO in the SI and colon slides was performed with the investigator blinded to condition using MetaMorph software (MDS Analytical Technologies) and a digital color camera (Cool Snap, Roper Scientific) mounted on an Olympus BX50 fluorescence microscope (Olympus). Images were acquired at 40 × in a standard-sized region of 75.2 mm^2^ chosen were visually determined to have the highest density of MPO expression. The presence of a DAPI + nucleus in MPO + cells was required for a cell to be counted as a neutrophil^60,61^. To quantify ZO-1, sections from all mice were assayed together, single-channel images converted to greyscale, and ZO-1 + pixels thresholded using the Triangle auto threshold option in ImageJ (Version 1.51). Background integrated density was subtracted to determine the corrected integrated density of ZO-1 expression.

### Statistical analyses

Using SPSSv.23 mixed factorial analyses of variance (ANOVA) analyzed the main effects and interactions of the independent variables of LPS treatment (control, acute LPS, chronic LPS) and tastant stimulus concentration on the standardized lick ratios for brief-access behavioral tests. When a significant main effect of LPS treatment or an interaction between LPS treatment and stimulus concentration was identified, Bonferroni post-hoc t-tests were performed to identify if the LPS treatment affected all or specific concentrations of each tastant.

For the microstructural variables defined in the long-term behavior tests, Two-tailed, paired sample t-tests were used to determine significant effects of chronic LPS treatment compared to control for each tastant with only one concentration tested. A repeated measures ANOVA was used to analyze the effects of chronic LPS treatment across two concentrations of sucrose.

Neurophysiological responses to multiple stimulus concentrations were compared with mixed-model analyses with treatment and stimulus concentration as factors followed by Bonferroni multiple comparison tests using Prism 8.3.1 software. One-way ANOVAs with Bonferroni post-tests were to compare neural responses to single stimuli. Body weight and food intake were compared between chronically gavaged LPS or water groups using repeated-measures ANOVAs with time and treatment as factors followed by Bonferroni multiple comparison tests. Two-tailed, unpaired Student’s t-tests were used to compare the number of MPO + neutrophils / standard area, ZO-1 expression, and taste gene expression in control and LPS-treated groups. Welch’s correction was used for ZO-1 t-tests since the variance was unequal. A criterion of *p* < 0.05 was used to report statistically significant results for all analyses.

## Supplementary information

Supplementary Legends.

Supplementary Figure S1.

Supplementary Figure S2.
