# Supplementary material for: Behavioral and neurophysiological taste responses to sweet and salt are diminished in a model of subclinical intestinal inflammation
Source: Sci Rep. 2020 Oct 19;10:17611. doi: 10.1038/s41598-020-74632-6 (PMC7573616; doi:10.1038/s41598-020-74632-6)
Supplement: Supplementary file 1 — Supplementary Legends. [file 41598_2020_74632_MOESM1_ESM.docx]

**SUPPLEMENTARY INFORMATION**

Behavioral and neurophysiological taste responses to sweet and salt are diminished in a model of subclinical intestinal inflammation

David W. Pittman^1^, Guangkuo Dong^2^, Alexandra M. Brantly^1^, Lianying He^3^, Tyler S. Nelson^1^, Schuyler Kogan^2^, Julia Powell^2^ and **Lynnette Phillips McCluskey^2^

Figure S1. Average standardized lick ratios (± S.E.M.) during brief-access (30 s) trials for MSG (A rats n = 13 / group; B mice n = 8 / group), quinine (C rats; D mice) and NaCl (E rats).

Figure S2. Body weight and food intake over time following enteral treatment. There were no significant differences in (A) body weight or (B) food intake in mice gavaged weekly with LPS (n = 6) compared to water (n = 6), though there was a significant effect of time on body weight in both groups as described in the text. Arrows indicate days on which mice were gavaged.
