# Supplementary figures and images for: Behavioral and neurophysiological taste responses to sweet and salt are diminished in a model of subclinical intestinal inflammation
Source: Sci Rep. 2020 Oct 19;10:17611. doi: 10.1038/s41598-020-74632-6 (PMC7573616; doi:10.1038/s41598-020-74632-6)

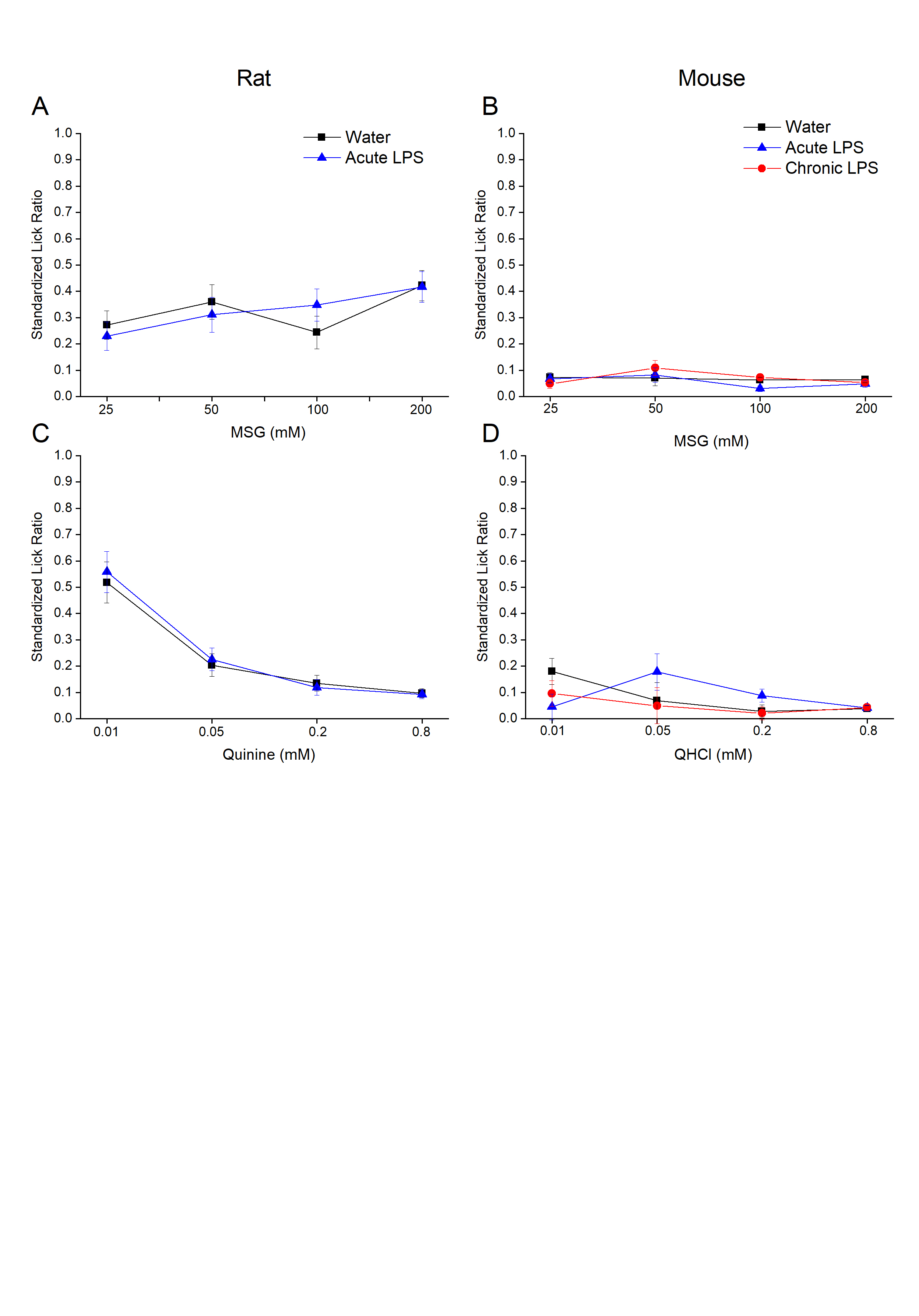

Supplement: Supplementary file 2 — Supplementary Figure S1. [file 41598_2020_74632_MOESM2_ESM.jpg]

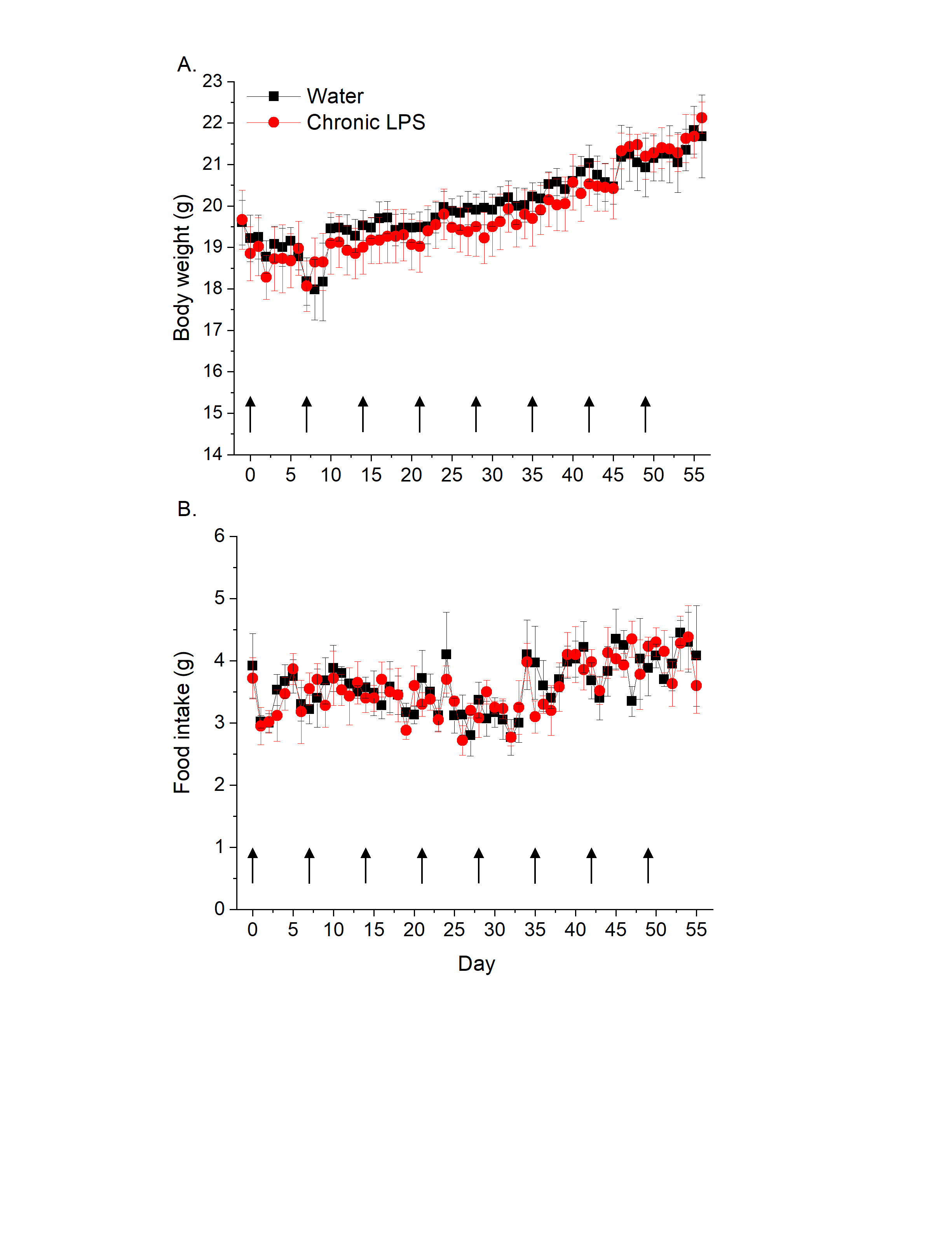

Supplement: Supplementary file 3 — Supplementary Figure S2. [file 41598_2020_74632_MOESM3_ESM.jpg]
